# Effect of Foot Insole on Planter Pressure Distribution in Patients with Neuropathic Diabetic Foot Ulcer: A Prospective, Randomized, Double-Blinded, Controlled Clinical Trial

**DOI:** 10.3390/medicina60122066

**Published:** 2024-12-16

**Authors:** Hany M. Elgohary, Ibtsam Allam, Ahmed M. N. Tolba, Faten Ali, Reem M. Alwhaibi, Hoda M. Zakaria, Walaa M. Ragab, Youssef Elbalawy

**Affiliations:** 1 Physical Therapy Department, Faculty of Applied Medical Sciences, Jerash University, Jerash 26110, Jordan; hmielgohary@gmail.com; 2 Department of Physical Therapy for Surgery, Faculty of Physical Therapy, Cairo University, Cairo 12613, Egypt; 3 Department of Physical Therapy for Skin and Surgery, Faculty of Physical Therapy, Suez Canal University, Ismailia 41522, Egypt; ebtsam_allam@pt.suez.edu.eg; 4 Department of Physical Therapy for Skin and Surgery, Faculty of Physical Therapy, Menoufia National University, Birket el Sab 32651, Egypt; 5 Department of Basic Science, Faculty of Physical Therapy, Delta University for Science and Technology, Gamasa 35511, Egypt; ahnasr01001897666@gmail.com; 6 Department of Physical Therapy for Internal Medicine and Geriatrics, Faculty of Applied Medical Science, Al al-Bayt University, P.O. Box 130040, Mafraq 24113, Jordan; alifaten@rocketmail.com; 7 Department of Physical Therapy for Internal Medicine and Geriatrics, Faculty of Physical Therapy, Delta University for Science and Technology, Gamasa 35511, Egypt; 8 Rehabilitation Sciences Department, Health and Rehabilitation Sciences College, Princess Nourah Bint Abdulrahman University, Riyadh 11671, Saudi Arabia; rmalwhaibi@pnu.edu.sa; 9 Department of Physical Therapy for Neurology and Neurosurgery, Faculty of Physical Therapy, Cairo University, Cairo 12613, Egypt; dr_hodazakaria@yahoo.com (H.M.Z.); youssef.mohamed@pt.cu.edu.eg (Y.E.); 10 Department of Physical Therapy, College of Medical Rehabilitation Sciences, Taibah University, Medina 42353, Saudi Arabia; 11 Department of Physical Therapy for Neurology, Faculty of Physical Therapy, Menoufia National University, Menoufia 32651, Egypt

**Keywords:** foot insole, peak pressure, pressure–time integral, polyneuropathy diabetic foot ulcer, pressure distribution

## Abstract

*Background and Objectives:* Patients with diabetes polyneuropathy are at a heightened risk for developing foot ulcers, often due to dynamic plantar foot pressure patterns that lead to increased pressure and shear forces in specific foot areas. This study aimed to evaluate the effects of foot insoles on peak pressure and the pressure–time integral in patients with polyneuropathy diabetic foot ulcers over a twelve-week period followed by an eight-week follow up. *Materials and Methods*: This was a prospective, randomized, double-blinded, controlled clinical trial involving 60 patients aged between 50 and 65 years of both genders. Inclusion criteria included midfoot ulcer grades II or III, a history of polyneuropathy diabetic foot ulcers lasting between six months and one year, diabetes duration of seven to ten years, glycated hemoglobin levels between 7% and 9%, and a body mass index (BMI) ranging from 25 to 30 kg/m^2^. Participants were randomly assigned to either the study group, which received foot insoles along with medication and wound care, or the control group, which received only medication and wound care. Measurements of peak pressure and pressure–time integrals were taken at the start of this study, after twelve weeks, and again eight weeks post-study. *Results*: The results indicated significant differences in peak pressure and pressure–time integral measurements for the rearfoot, midfoot, hallux, and both medial and lateral forefoot areas after twelve weeks of using foot insoles compared to the control group. This suggests that the use of foot insoles effectively reduces peak pressure and the pressure–time integral in these critical areas. *Conclusions*: The findings of this study support the use of foot insoles as a beneficial intervention for decreasing peak pressure and the pressure–time integral on the hallux, medial, and lateral forefoot in patients with polyneuropathic diabetic foot ulcers, and they could play a crucial role in preventing further complications.

## 1. Introduction

A persistent, non-healing foot ulcer represents a significant health concern for individuals with polyneuropathy diabetes mellitus [1]. The prevalence of diabetic foot ulcers among diabetic polyneuropathic patients varies widely, with studies in Egypt reporting rates between 6.1% and 29.3% [2,3]. Chronic wounds, such as foot ulcers, impose considerable financial and health burdens on both individuals and society [4]. Notably, foot ulcers are responsible for over 85% of lower limb amputations, and diabetes is a leading cause of non-traumatic amputations worldwide, and it is found to be up to 15 times higher than in non-diabetic populations [5,6].

Several factors contribute to the onset of foot ulcers, including neuropathy as sensory impairment, which is the main cause of foot ulcers and affects weight distribution on the foot, peripheral vascular disease, a history of previous ulcers or amputations, skin conditions, and excessive plantar pressure resulting from foot deformities. The use of appropriate footwear has been demonstrated to significantly reduce the risk of foot ulcers among the 347 million individuals worldwide living with diabetic polyneuropathy [7].

To better understand how the redistribution of plantar pressure through insoles can influence symptoms, assessments of regional plantar pressure distribution can be beneficial. Research indicates that using contoured insoles can shift peak pressures from the rearfoot to the midfoot in patients experiencing foot pain due to prolonged standing, leading to a reduction in pain, discomfort, and fatigue. This suggests that insoles not only alleviate symptoms but also play a crucial role in managing foot health for those at risk of ulcers [8].

Research on offloading interventions for treating neuropathic ulcers, particularly those complicated by infection or ischemia, as well as non-plantar and plantar heel ulcers, remains relatively sparse [9]. Previous studies have indicated that wearing specialized shoes equipped with custom-made or molded insoles can effectively reduce high plantar pressure [10].

The primary objective of the current study was to assess the changes in peak pressure and the pressure–time integral after twelve weeks of using foot insoles, followed by an eight-week follow-up period in patients with polyneuropathy diabetic foot ulcers. This investigation aims to provide insights into the effectiveness of insoles in managing pressure distribution and enhancing healing outcomes for individuals suffering from polyneuropathic diabetic foot complications.

## 2. Materials and Methods

### 2.1. Subjects

The clinical trial was designed as a randomized, double-blind controlled study involving both patients and assessors. Participants were recruited from Damietta General Hospital, and the clinical applications of foot insoles, along with physical assessments, were conducted at motion analysis laboratories within the Faculty of Physical Therapy at Delta University for Science and Technology between September 2021 and May 2022.

This study received approval from the Ethical Committee for Human Research at Delta University’s Faculty of Physical Therapy (reference number F.P.T 2207014; Ethical Approval Date: May 2021). Per the principles outlined in the 1975 Helsinki Declaration, all patients were invited to participate and provided written informed consent before enrollment. Additionally, this study was registered on ClinicalTrials.gov under the identifier NCT05888259.

Patients diagnosed with diabetes mellitus (type II) having midfoot ulcers were referred to physical therapy by either a general surgeon or a general practitioner. To be included in this study, participants had to meet specific criteria: they needed to be between 50 and 65 years old of both genders without any history of receiving foot insoles and with midfoot ulcer grades II or III according to the Wagner classification system [5]. Additionally, they should have had neuropathic diabetic foot ulcers for a duration of 6 months to 1 year; a comprehensive screening for neuropathy to confirm appropriate participant inclusion was assessed utilizing a tuning fork test to evaluate vibratory sensation at the great toe with a 128 Hz tuning fork. Also, a history of diabetes mellitus (type II) lasting between seven and ten years, glycated hemoglobin levels between 7% and 9%, and a body mass index (BMI) ranging from 25 to 30 kg/m^2^ were included in this study. Exclusion criteria included the presence of Raynaud’s disease, peripheral arterial disease, foot deformities, burns on the feet, or lower limb anesthesia. Patients who declined to participate or did not sign the informed consent forms were also excluded from this study.

### 2.2. Sample Size

The sample size for this study was determined using G*Power 3.1 software to detect a 40% difference in the dynamic plantar pressure gradient. A 95% confidence interval was established, allowing for a 5% error margin. Consequently, the estimated sample size was set at 28 patients per group, which was increased by 15% to 32 patients to accommodate potential dropouts from randomization through the completion of the treatment protocol.

Participants were randomly assigned to either the control group, which received only medication and wound care plus wearing standard footwear without foot insoles, or the study group, which received foot insoles along with the same medication and wound care.

Medication for both groups includes Metformin at a dosage of 500 mg, taken twice daily, to manage blood sugar levels. They also received Gabapentin at a dosage of 300 mg once daily to alleviate neuropathic pain. To prevent infections, participants were treated with Amoxicillin–Clavulanate, prescribed at 875 mg/125 mg twice daily.

Wound care for both groups includes hydrocolloid dressings that were changed every 3 to 5 days or as necessary to maintain a moist healing environment. Additionally, silver sulfadiazine cream was applied in a thin layer once daily for its antimicrobial benefits, and sharp debridement was carried out as needed, typically every 1 to 2 weeks.

This randomization was achieved using a computer-generated random number table and sealed opaque envelopes, with the process managed by authors who were not involved in data collection. All patients underwent assessments using a system that included the FREE MED platform and CLINICAL 3DMA. Outcomes were measured at baseline, at the end of the 12-week intervention, and again 8 weeks post-intervention for follow up. A senior researcher, who was unaware of the group assignments and considered part of the research team, collected all outcome measures.

### 2.3. Procedure

The assessment platform consists of two blocks positioned side by side, each measuring 100 × 62 cm. Calibration was conducted with a 10-bit auto setting, an XY resolution of 2.5 dpi, a Z resolution of 8 bits, a maximum pressure capacity of 150 N/cm^2^, and a frequency of 120 Hz.

To ensure accurate measurements, the platform was placed on a flat, hard, clean, and regular surface. It was positioned within the detection area of the CLINICAL 3-DMA system, which was marked off with tape to define the boundaries. Special attention was given to the setup to maintain consistency and reliability in the data collected during this study.

### 2.4. Preparation

The air conditioning was maintained at 28 degrees Celsius to ensure a comfortable room temperature during the evaluations. Calibration of the CLINICAL 3DMA system was confirmed by positioning the wand vertically in the center of the detection area, ensuring it was free from any obstructions or markings prior to each session. If the calibration results were satisfactory, the evaluation session commenced; otherwise, recalibration was performed.

Demographic information, including gender, name, age, body mass index, and height, was recorded in both the CLINICAL 3DMA and FREE STEP 7 software systems. Participants were instructed to walk within the detection field of the CLINICAL 3DMA system using the FREE STEP platform [11]. They were guided to start, stop, walk out of the detection area, and then re-enter the platform. Participants were encouraged to become comfortable within the capture field to ensure accurate data collection. It was emphasized that they should walk at their normal, steady pace, as variations in walking speed could significantly influence the recorded outcomes.

After participants acclimated to the testing environment, a 1 min recording session was initiated. Upon successful completion of the recording, the data were saved to the designated computer for further analysis.

### 2.5. Data Processing

Data collected from the CLINICAL 3DMA and FREE STEP software were selected after completing the recording session. Only complete footprints that were fully contained within the sensory area of the platform were considered valid; any partial footprints (either the first or last) were excluded using the footprint selection tool to maintain accuracy.

Pressure maps were generated to assess peak pressures and pressure–time integrals across various foot regions, including the hindfoot, midfoot, hallux, medial forefoot, and lateral forefoot. The measurements were then uploaded to a server that utilizes advanced technology to create a 3D model of the foot insole based on multiple images captured by the user.

The 3D scan data was subsequently transferred to EasyCAD 2 software, where the model was adjusted to accurately reflect the subject’s foot geometry. This modified model was then imported into a 3D printing device. For participants with foot ulcers, the insoles were specifically designed to alleviate pressure on the ulcerated area. Each participant utilized their custom foot insole for a duration of 12 weeks before the evaluation of study outcomes.

### 2.6. Insole Design

Each insole was custom designed for the individual participants to ensure the best fit and support. Molds were taken from the participants’ feet to create these tailored insoles. The molding process utilized a flexible material that accurately captured the foot’s contours, allowing for an exact fit. This approach ensured that the insoles offered the necessary support and comfort, customized to each participant’s unique foot structure.

The foot insole features a thickness of 5 mm beneath the flat section of the forefoot, with modifications achieved by adjusting the position of the metatarsal bar, which adds 5 mm above this flat area. The placement of the metatarsal rod and the large cavity distal to it were determined based on the distribution of plantar pressure.

The positioning and contour of the distal end of the metatarsal bar were defined by a line corresponding to the area where the plantar pressure reached 75% of the maximum value. This line also indicated the proximal boundary of the void. The distal edge of the void was located near the peak plantar pressure area, where the pressure was 10% of the maximum peak pressure, resulting in a void depth of 3 mm.

**Figure 1:**
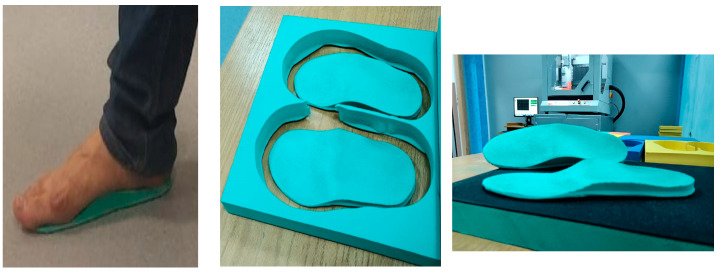
Foot insole processing.

Following the initial design of the metatarsal bars and the void, two variations were developed by adjusting the metatarsal bar’s position either proximal or distal by 2–4% of the insole length, depending on the location of the foot ulcer. Based on prior pilot study findings, this adjustment corresponds to a 5–7 mm shift on a size 38 orthotic insole. The orthotic insoles were crafted from medium-density ethylene-vinyl acetate (EVA) with a hardness of 50° Shore A, utilizing a CNC machine for manufacturing precision (Figure 1). 

To be sure that the study group wore insoles correctly, we implemented several strategies. First, we conducted an initial training session for all participants, providing them with comprehensive instructions and demonstrations on how to properly use the insoles. Additionally, we scheduled regular follow-up sessions, such as weekly check ins, to remind participants of the importance of adherence and to address any issues they faced. We also supplied usage logs for participants to document their daily wear time, which helped us monitor consistency. Furthermore, we established a feedback mechanism that enabled participants to report any discomfort or difficulties with the insoles, allowing us to respond quickly and ensure proper usage. To measure adherence to insole usage in our study, participants wore the insoles for a minimum of 6 h per day for 5 days per week, providing participants with a specific target to work towards.

### 2.7. Statistical Analysis

Statistical analysis was conducted using SPSS software for Windows, version 21.0 (Chicago, IL, USA). Descriptive statistics were computed for both groups at three different time points: at baseline, after twelve weeks, and again eight weeks later during the follow up. To assess the normality of the data distribution, the Shapiro–Wilk test was employed. Differences in mean change scores for peak pressure and the pressure–time integral were analyzed using Student’s *t*-test, a one-way ANOVA, and a two-way mixed-model ANOVA. A significance level of *p* > 0.05 was established to determine any statistically significant differences between and within the groups at each time interval.

## 3. Results

**Figure 2:**
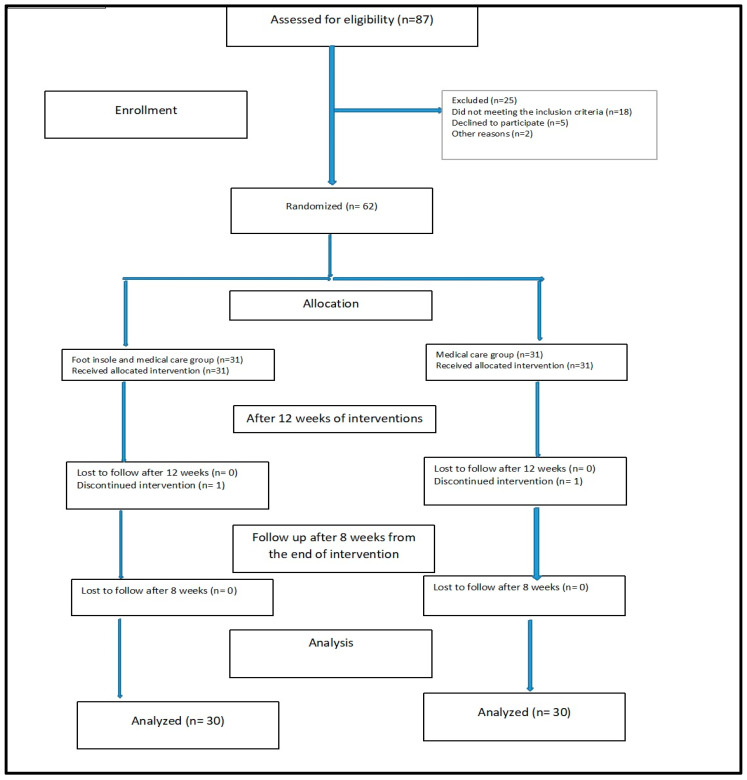
Flowchart of the patients.

The patient flowchart throughout this study is illustrated in Figure 2. A total of eighty-seven patients with polyneuropathic diabetic foot ulcers classified as grades II and III were referred from Damietta General Hospital to the outpatient clinic at the Faculty of Physical Therapy, Delta University for Science and Technology, for eligibility assessment. Out of these, eighteen patients were excluded for not meeting the inclusion criteria, five declined to participate, and two were removed from this study; the reasons for their withdrawal were not linked to this study’s interventions or outcomes. Consequently, sixty-two patients were deemed eligible to take part in the trial and were randomly assigned to two equal groups. However, due to transportation issues, two patients, one from the foot insole and medical care group and one from the medical care group, were unable to complete the trial.

Throughout this study, no patients in either the foot insole or medical care groups reported any adverse effects. The baseline data regarding the bio-demographic and clinical characteristics of all participants—including age, body mass index (BMI), sex distribution, duration since the onset of diabetic polyneuropathic foot ulcers, ulcer grades, walking speed on the force platform, peak pressure across various foot areas, and pressure–time integral—showed no statistically significant differences between the two groups (Table 1).

When analyzing the peak pressure and the pressure–time integral among patients in the foot insole group across three measurement points (pre, post I, and post II), a one-way ANOVA with repeated measures indicated a statistically significant difference (*p* < 0.05). In contrast, the medical care group did not show any statistically significant differences for the same parameters (*p* > 0.05) (Table 2).

To evaluate the differences between the initial measurements and the endpoints (pre vs. post I and pre vs. post II) for groups A and B, a one-way ANOVA followed by a post hoc Tukey test was conducted. This analysis focused on peak pressure and the pressure–time integral across various foot regions, including the rearfoot, midfoot, hallux, medial forefoot, and lateral forefoot (Table 3 and Table 4).

The two-way ANOVA (2 × 3 mixed-model) analysis demonstrated that the results for peak pressure and the pressure–time integral across the rearfoot, midfoot, hallux, medial forefoot, and lateral forefoot exhibited statistically significant differences. These differences were observed when comparing values at baseline, after 12 weeks of intervention, and 8 weeks post-intervention during the follow up. The specific *p-* and F-values are detailed in Table 5.

## 4. Discussion

The primary outcomes of this randomized controlled study indicated that the use of foot insoles significantly reduced peak pressure and the pressure–time integral in the medial forefoot, lateral forefoot, and hallux areas. These improvements were observed after twelve weeks of using the insoles and continued to be evident eight weeks after the intervention ended, with a significance level of *p* < 0.05.

Concerns have been raised regarding the potential long-term effects of the insoles on patients’ loading patterns on the plantar surface of the foot, particularly given that the control group did not show any improvement in their measurements during the same timeframe. Importantly, no adverse effects were reported by any patients in the experimental group while using the foot insoles.

Research has consistently indicated that plantar ulcers often precede lower extremity amputations, particularly in individuals with diabetes. Elevated plantar pressure is a significant contributor to the formation of these ulcers. Additionally, various structural abnormalities in the foot have been associated with increased concentrations of plantar pressure, which can exacerbate the risk of ulceration [12].

The specific design elements and materials that can effectively reduce plantar pressure in the forefoot region have not yet been clearly defined [13]. However, it is well established that lowering plantar pressure significantly decreases the risk of ulcer formation and recurrence. Previous research has indicated that a custom-molded orthosis, particularly when modified, is more effective in managing plantar pressure compared to a standard custom-molded orthosis without modifications. This suggests that tailored interventions can play a crucial role in preventing complications associated with elevated plantar pressure [14].

Insole modifications can include various features designed to alleviate plantar pressure in the forefoot region. Examples of these modifications are local cushioning, replacing top coverings with plastazote, adjusting or adding metatarsal bars and arches, removing unnecessary holes, and incorporating arch supports [15,16,17].

In this study, the non-affected foot model was created without any modifications, while the subject-specific insole was customized to alleviate pressure at the ulcer site. After 3D printing, the insole was tested by placing it into the subject’s shoe, specifically for cases involving foot ulcers. This tailored adjustment significantly reduced both plantar pressure and the pressure–time integral at the distal portion of the metatarsal bar, directly where the ulcer was located. By repositioning the metatarsal bar and creating a void, the design aimed to enhance blood flow to the forefoot, potentially facilitating the healing process of the ulcer.

Products take into account the structural alterations that develop in the diabetic foot, but personalized devices reduce plantar pressure and PTI more than prefabricated ones, which indicates that they may be preferred in practice [18,19]. Other studies that examined the efficacy of customizable and prefabricated insoles to reduce PPP without accounting for cost showed that customized insoles were superior at offloading pressure in approximately every location of the foot [20]. A handful of other research provided support for the findings of the present research. Custom-molded foot orthoses, according to research by Altayyar, can increase total contact area by 5–10% while lowering peak plantar pressure by 30–40% [21].

Research conducted by Lee P.Y. et al. in 2014 compared the clinical effectiveness of using forefoot cushions versus a shoe-only condition. The study found that forefoot cushions significantly decreased both peak pressure and maximum force in the forefoot. Specifically, the plantar shield and metatarsal dome, positioned 5 mm distal to the metatarsal heads, resulted in reductions of 17% and 19% in peak pressure, respectively, with a significance level of *p* < 0.001. These results suggest that when addressing forefoot pressures in older adults experiencing forefoot pain, the position of the cushioning relative to the metatarsal heads may be more critical than the specific design of the cushioning itself [22].

The rationale for the current study is supported by previous research, demonstrating that offloading is a vital therapeutic approach in managing diabetic foot ulcers. According to de Oliveira and Moore (2015), total contact casts (TCCs) have emerged as one of the most effective devices for promoting ulcer healing. However, these casts are not without their complications, and there is limited understanding of their impact on costs, patient compliance, and quality of life. This highlights the need for further investigation into these aspects to optimize treatment strategies for diabetic foot ulcers [23].

According to the findings of Martinez-Santos et al. in 2019, the effects of offloading were consistent regardless of the material used, with significant reductions in pressure occurring when the anterior edge of the metatarsal bar was positioned at 77% of the peak pressure values whether using EVA or Poron materials. Among the nine different orthotic insoles evaluated, those using the optimal design resulted in 51% of participants experiencing pressure exceeding 200 KPa in one or more regions of the metatarsal heads. In contrast, 61% of individuals using a flat insole and 58% using standard orthotic design criteria reported similar pressure levels. This indicates that the design and positioning of the metatarsal bar are crucial for effective pressure management in the forefoot [24].

Insoles featuring a modified metatarsal bar may play a significant role in preventing or treating ulcers in the hallux and forefoot regions, provided that adequate pressure is maintained in other areas of the foot. However, further research is necessary to fully understand the effects and benefits of these modifications on ulcer management and overall foot health [25,26].

The current study has several limitations that should be acknowledged. Firstly, although the patient population was limited, this study successfully identified statistically significant differences in peak pressure and the pressure–time integral, with changes ranging from 29% to 33%. This suggests a need for further research involving a larger sample size to validate these findings.

Secondly, the duration of this study, which involved a twelve-week period of foot insole usage, may not be sufficient to assess the long-term effectiveness of these insoles in managing diabetic foot ulcers. More extensive studies are needed to evaluate their impact over a longer timeframe. Additionally, the research did not establish a clear link between the effectiveness of foot insoles and the recovery or prevention of foot ulcers. Understanding how these insoles influence blood circulation and the subsequent healing of ulcers requires further investigation. Moreover, it is crucial to focus on preventing initial neuropathic plantar forefoot ulcers rather than merely addressing their recurrence.

Lastly, there is a notable gap in the literature regarding patient expectations, effective footwear education, and the design of activity-specific devices. The studies also considered geographic and socioeconomic factors, which may influence the outcomes and applicability of the findings. In addition, collecting detailed retrospective data on footwear in future studies will enable more comprehensive comparisons, particularly since a limitation of our study is the age of the selected patients. To enhance generalizability, future research should expand the age range and include individuals with other comorbidities, such as hypertension. Additionally, it is recommended to incorporate further outcome measures, such as ulcer healing rates, pain intensity, and quality of life.

## 5. Conclusions

A useful technique to alter the plantar pressure distribution is a foot insole with modified metatarsal bars, which may aid in the healing of diabetic foot ulcers. This alters the pressure–time integral and the plantar pressure distribution.

## Figures and Tables

**Table 1:** The baseline characteristics of patients for both groups A and B.

		**Group A** **(Mean ± SD)**		**Group B** **(Mean ± SD)**		***p*** **-Value**
**Age (years)**		58.10 ± 4.60		58.30 ± 4.20		0.86
**BMI (kg/m^2^)**		27.20 ± 1.62		27.34 ± 1.51		0.74
**HBA1C (%)**		7.74 ± 0.66		7.870 ± 0.649		0.44
**Duration of diabetic foot (month)**		8.90 ± 2.06		8.833 ± 1.821		0.89
**The velocity of walking (meters/min.)**		63.20 ± 4.92		61.86 ± 5.56		0.31
**Male/female (%)**		(15/15) (50%/50%)		(16/14) (53.33%/46.67%)		
**Grades of ulcer (II or III)**		(15/15) (50%/50%)		(17/13) (56.67%/43.33%)		
	**Peak Pressure**			**Pressure–Time Integral**		
	**Group A (Pre)**	**Group B (Pre)**	***p*-Value**	**Group A (Pre)**	**Group B (Pre)**	***p*-Value**
**Rearfoot**	416.50 ±26.07	423.50 ±31.93	0.36	106.17 ±24.17	102.83 ±22.16	0.58
**Midfoot**	236.17 ±50.22	239.17 ±51.48	0.82	73.67 ±22.70	71.50 ±21.22	0.70
**Hallux**	329.17 ±26.10	331.33 ±23.89	0.74	62.63 ±12.57	64.30 ±13.42	0.62
**Medial forefoot**	420.83 ±27.54	408.33 ±22.53	0.06	106.17 ±17.94	102.00 ±19.94	0.39
**Lateral forefoot**	426.33 ±32.61	429.33 ±31.42	0.72	120.83 ±21.94	127.17 ±23.44	0.28

SD: standard deviation; *p*-value: probability value; BMI: body mass index; HBA1C: hemoglobin A1C (HbA1c) test.

**Table 2:** One-way ANOVA repeated measures for peak pressure and the pressure–time integral.

	Group A (Mean ± SD)				Group B (Mean ± SD)			
	Pre	Post I	Post II	*p*-Value	Pre	Post I	Post II	*p*-Value
**Peak Pressure**								
**Rearfoot**	416.50 ±26.07	615.33 ±33.19	628.83 ±28.58	<0.0001 *	423.50 ±31.93	417.17 ±21.76	430.83 ±27.64	0.79
**Midfoot**	236.17 ±50.22	356.67 ±23.28	376.50 ±31.33	<0.0001 *	239.17 ±51.48	239.17 ±36.65	243.33 ±43.65	0.54
**Hallux**	329.17 ±26.10	218.83 ±26.93	209.67 ±27.07	<0.0001 *	331.33 ±23.89	334.50 ±24.12	333.17 ±23.51	0.65
**Medial forefoot**	420.83 ±27.55	279.83 ±31.77	269.83 ±32.81	<0.0001 *	408.33 ±22.53	410.83 ±29.95	409.83 ±22.99	0.75
**Lateral forefoot**	426.33 ±32.61	283.83 ±36.92	277.67 ±35.64	<0.0001 *	429.33 ±31.42	425.50 ±28.48	423.67 ±27.16	0.48
**Pressure–Time Integral**								
**Rearfoot**	106.17 ±24.17	154.50 ±8.55	163.33 ±12.34	<0.0001 *	102.83 ±22.16	103.00 ±16.90	103.83 ±18.96	0.93
**Midfoot**	73.67 ±22.70	109 ±23.36	116.33 ±20.13	<0.0001 *	71.50 ±21.22	74.83 13.29	74.667 ±15.13	0.41
**Hallux**	62.63 ±12.57	60.93 ±13.02	57.03 ±12.83	<0.0001 *	64.30 ±13.42	63.27 ±17.69	62.367 ±17.07	0.75
**Medial forefoot**	106.17 ±17.94	101.83 ±16.21	97.17 ±16.75	<0.0001 *	102.00 ±19.94	99.33 ±21.96	100.667 ±18.27	0.23
**Lateral forefoot**	120.83 ±21.94	117.17 ±19.55	168.83 ±17.20	<0.0001 *	127.17 ±23.44	125.83 ±19.74	124.50 ±18.39	0.46

SD: standard deviation; *p*-value: probability value; * statistically significant.

**Table 3:** Mean difference and confidence interval (95%) using a one-way ANOVA with a post hoc Tukey test for the pressure–time integral in groups A and B.

		Group A			Group B		
	Time	MD	Confidence Interval		MD	Confidence Interval	
			Lower	Upper		Lower	Upper
**Rearfoot**	Pre × Post I	198.83	180.72	216.95	−6.33	−23.22	10.55
	Pre *×* Post II	212.33	194.22	230.45	7.33	−9.55	24.22
	Post I *×* Post II	13.5	−4.62	31.62	13.67	−3.21	30.55
**Midfoot**	Pre × Post I	120.50	97.89	143.11	3.33	−22.54	29.21
	Pre *×* Post II	140.33	117.73	162.99	4.16	−21.45	29.78
	Post I *×* Post II	19.83	−2.77	42.44	4.16	−21.45	29.78
**Hallux**	Pre × Post I	3.17	−12.67	18.51	3.16	−11.51	17.84
	Pre *×* Post II	−121.67	−137.099	−106.235	1.83	−12.84	16.51
	Post I *×* Post II	−124.83	−140.26	−109.40	1.33	−16.01	13.34
**Medial forefoot**	Pre × Post I	−141.00	−159.79	−122.24	2.50	−13.13	18.13
	Pre *×* Post II	−155.5	−174.21	−136.71	1.50	−14.13	17.13
	Post I *×* Post II	−14.50	−33.29	−4.29	1.00	−16.63	14.63
**Lateral forefoot**	Pre × Post I	−142.50	−164.11	−120.88	−3.83	−21.73	14.07
	Pre *×* Post II	−148.67	−170.29	−127.5	−5.67	−23.57	12.23
	Post I *×* Post II	−6.17	−27.78	15.46	−1.83	−19.72	16.07

MD: mean difference.

**Table 4:** Mean difference and confidence interval (95%) of the pressure–time integral for groups A and B.

		Group A			Group B		
	Time	MD	Confidence Interval		MD	Confidence Interval	
			Lower	Upper		Lower	Upper
**Rearfoot**	Pre × Post I	48.33	38.22	58.45	0.167	−11.83	12.17
	Pre × Post II	57.17	47.05	67.28	1.00	−10. 98	12.98
	Post I × Post II	8.83	−1.28	18.95	0.83	−11.17	12.83
**Midfoot**	Pre × Post I	35.33	21.72	48.94	4.33	−6.98	15.63
	Pre × Post II	42.67	29.06	56.28	9.83	−1.47	21.13
	Post I × Post II	7.33	−6.28	20.94	5.50	−5.80	16.80
**Hallux**	Pre × Post I	−1.7	−9.58	6.18	−1.03	−10.99	8.92
	Pre × Post II	−5.6	−13.48	2.28	−1.93	−11.89	8.02
	Post I × Post II	−3.9	−11.78	3.94	−0.9	−10.86	9.06
**Medial forefoot**	Pre × Post I	−4.33	−14.79	6.12	−2.67	−15.05	9.72
	Pre × Post II	−9.00	−19.46	1.46	−1.33	−13.72	11.05
	Post I × Post II	−4.67	−15.12	5.79	1.33	−11.05	13.72
**Lateral forefoot**	Pre × Post I	−3.67	−15.77	8.44	−1.33	−14.04	11.37
	Pre × Post II	−11.00	−23.10	1.10	−2.67	−15.37	10.04
	Post I × Post II	−7.33	−19.44	4.77	−1.33	−14.04	11.37

MD: mean difference.

**Table 5:** Two-way 2 × 3 mixed model showing *p-* and F-values for peak pressure and the pressure–time integral.

	Peak Pressure		Pressure–Time Integral	
	*p*-Value	F-Value	*p*-Value	F-Value
**1—Rearfoot**				
**Within subjects**	<0.0001 *	935.91	<0.0001 *	201.23
**Between subjects**	<0.0001 *	265.87	<0.0001 *	44.87
**Interaction**	<0.0001 *	259.91	<0.0001 *	42.63
**2—Midfoot**				
**Within subjects**	<0.0001 *	191.67	<0.0001 *	59.92
**Between subjects**	<0.0001 *	58.82	<0.0001 *	27.22
**Interaction**	<0.0001 *	52.38	<0.0001 *	12.37
**3—Hallux**				
**Within subjects**	<0.0001 *	454.55	0.048 *	3.98
**Between subjects**	<0.0001 *	99.28	0.88	0.13
**Interaction**	<0.0001 *	107.96	0.28	1.29
**4—Medial forefoot**				
**Within subjects**	<0.0001 *	439.02	0.70	0.15
**Between subjects**	<0.0001 *	136.90	0.30	1.20
**Interaction**	<0.0001 *	144.15	0.50	0.70
**5—Lateral forefoot**				
**Within subjects**	<0.0001 *	466.62	<0.0001 *	10.84
**Between subjects**	<0.0001 *	109.00	0.17	1.77
**Interaction**	<0.0001 *	95.53	0.51	0.68

* Statistically significant; *p*-value: probability value; F-value: null distribution.

## Data Availability

The data can be requested from the corresponding author and will be released upon reasonable request.
